# Yokukansan for perioperative psychiatric symptoms in cancer patients undergoing high invasive surgery. J-SUPPORT 1605 (ProD Study): study protocol for a randomized controlled trial

**DOI:** 10.1186/s13063-019-3202-1

**Published:** 2019-02-08

**Authors:** Saho Wada, Ryoichi Sadahiro, Yutaka J. Matsuoka, Yosuke Uchitomi, Takuhiro Yamaguchi, Ken Shimizu

**Affiliations:** 1 Department of Psycho-Oncology, National Cancer Center Hospital, 5-1-1, Tsukiji, Chuo-ku, Tokyo, Japan; 2 Division of Health Care Research, Behavioral Sciences and Survivorship Research Group, Center for Public Health Sciences, National Cancer Center Japan, 5-1-1, Tsukiji, Chuo-ku, Tokyo, Japan; 3 Innovation Center for Supportive, Palliative and Psychosocial Care, National Cancer Center Hospital, 5-1-1, Tsukiji, Chuo-ku, Tokyo, Japan; 4 Behavioral Sciences and Survivorship Research Group, Center for Public Health Sciences, National Cancer Center Japan, 5-1-1, Tsukiji, Chuo-ku, Tokyo, Japan; 5 Division of Biostatistics, Tohoku University Graduate School of Medicine, 1-1 Seiryo-machi, Aoba-ku, Sendai, Miyagi 980-8574 Japan

**Keywords:** Yokukansan, Oncology, Surgery, Anxiety, Delirium

## Abstract

**Background:**

Preoperative anxiety and postoperative delirium affect both short- and long-term prognoses in patients with cancer; therefore, these conditions require early prevention and treatment. However, no standard preventive or therapeutic methods have been established for them. Yokukansan, a Japanese herbal medicine for the treatment of insomnia and anxiety, causes relatively few adverse drug reactions and effectively improves the behavioral and psychological symptoms of dementia. Thus, it is expected to be useful for treating and/or preventing perioperative psychiatric symptoms in patients with cancer. The objective of this study is to clarify the therapeutic effect of Yokukansan for preoperative anxiety and its preventive effect on postoperative delirium in cancer patients, as well as to confirm its safety profile.

**Methods:**

This study is a randomized, double-blind, placebo-controlled study in cancer patients scheduled to undergo tumor resection. Patients who provide consent are randomly allocated to receive oral administration of Yokukansan or placebo, and study drug administration is continued for 4 days or longer prior to surgery. We defined two primary endpoints, change in preoperative anxiety and incidence of postoperative delirium. Secondary endpoints are severity score of postoperative delirium, duration of postoperative delirium, amount of benzodiazepines used prior to surgery, amount of antipsychotic agents used after surgery, and number of postoperative hospitalization days. We plan to complete the analysis on March 31, 2021. The target number of registered patients is 110 per group, or 220 in total.

**Discussion:**

This study is the first randomized, double-blind, placebo-controlled study intended to clarify the effects of a Japanese herbal medicine, Yokukansan, in the prevention and treatment of perioperative psychiatric symptoms in patients with cancer. The trial was initiated on August 14, 2017, with 195 subjects randomized by October 5, 2018.

**Trial registration:**

UMIN Clinical Trials Registry (UMIN-CTR), UMIN000027561. Registered on 31 May 2017.

**Electronic supplementary material:**

The online version of this article (10.1186/s13063-019-3202-1) contains supplementary material, which is available to authorized users.

## Background

Patients with cancer experience various psychiatric symptoms during the perioperative period. Anxiety not only lowers treatment compliance by impairing cancer patients’ decision-making ability [1], but it also exacerbates cancer symptoms, leading to reduced quality of life [2]. During the perioperative period, anxiety has also been reported to prolong postoperative pain [3], affect endocrine and immune functions, and influence the progression and long-term prognosis of cancer [4]. An observational study previously conducted at our hospital showed that preoperative anxiety occurred in approximately 15% of cancer patients [5]. Hence, we consider that reducing preoperative anxiety is an important goal of cancer treatment in general.

Delirium is a psychiatric disorder characterized by acute impairment of consciousness and lack of attention [6]. Postoperative delirium refers to delirium that occurs after patients recover from anesthesia, typically within 2 or 3 days after the operation [7–10]. Patients with postoperative delirium experience not only increased rates of respiratory infection and complications associated with immobility and prolongation of hospitalization, but also long-term adverse effects including increased mortality rate [11–13] and impaired cognitive function [14–16]. In the aforementioned observational study, postoperative delirium occurred in approximately 32% of cancer patients [5].

Perioperative psychiatric symptoms should be appropriately assessed, treated, and prevented. However, when used in patients with impaired metabolic capacity, benzodiazepines, which are common therapeutic agents for anxiety, may have long-lasting effects or cause muscle relaxation, resulting in falls. They are also considered to induce delirium when used in patients with cerebral vulnerability, including elderly patients and patients with dementia [17–21]. The antipsychotic agents used for the treatment of postoperative delirium may cause dose-dependent over-sedation or serious adverse drug reactions that include extrapyramidal symptoms and long QT syndrome. Moreover, antipsychotic agents have been reported to increase mortality risk when used in elderly patients with dementia and are often difficult to use in the treatment of elderly patients or patients with heart disease [22–24].

In these contexts, Yokukansan has attracted attention for its potential utility in the treatment of perioperative psychiatric symptoms in patients with cancer. Yokukansan is a Japanese herbal medicine for the treatment of insomnia and anxiety, and it effectively improves the behavioral and psychological symptoms of dementia (BPSD), including hallucinations, delusions, disinhibition, day/night reversal, wandering, irritability, verbal aggression, and risky behavior [25–29]. It has been hypothesized that Yokukansan may prevent postoperative delirium, which presents clinical symptoms very similar to those of BPSD [27, 30–34].

Several lines of empirical evidence imply that Yokukansan would be effective in this context. In a retrospective study (*n* = 19) in patients who underwent colorectal surgery, we found that the incidence of postoperative delirium after Yokukansan administration was lower than in previous studies [35]. In a pre/post study of patients who underwent gastrointestinal surgery (*n* = 8), oral administration of Yokukansan improved insomnia, dysesthesia, restless legs and behavior, and risky behavior [31]. In a randomized phase II trial in which patients underwent cardiovascular surgery (*n* = 30), Yokukansan significantly improved agitation, mood swings, deficient sense of reality, hallucinations, and delusions [33]. In another randomized phase II trial in patients undergoing surgery for gastrointestinal and lung malignancies (*n* = 186) [36], Yokukansan decreased the risk of postoperative delirium in patients with a Mini Mental State Examination score ≤ 26.

Adverse drug reactions, including hypokalemia, have been observed following the use of Yokukansan, but their incidence was no higher than 1.3% (41 of 3141 patients) [37, 38]. On the other hand, benzodiazepines and antipsychotic agents commonly used for these symptoms can cause serious adverse reactions. Although the effect of Yokukansan on anxiety and delirium is less certain than that of benzodiazepines and antipsychotic agents, major adverse events have not been observed following Yokukansan administration, suggesting that this herbal medication may have a major advantage in regard to safety.

Against this background, we planned a randomized controlled study on the efficacy of Yokukansan for perioperative psychiatric symptoms. The objective of this study is to clarify the therapeutic effect of Yokukansan for preoperative anxiety, its preventive effect on postoperative delirium, and its safety in patients with cancer.

## Methods

### Study design

The therapeutic effect of Yokukansan for preoperative anxiety, its preventive effect on postoperative delirium, and its safety in cancer patients will be confirmed in a randomized, double-blind, placebo-controlled study. The investigators registered this study in the UMIN Clinical Trials Registry (UMIN-CTR) https://upload.umin.ac.jp/cgi-open-bin/ctr/ctr_view.cgi?recptno=R000031488 (UMIN000027561) prior to the start of the trial.

### Study setting and sample

We are conducting our study at the National Cancer Center Hospital, a tertiary hospital for cancer in Japan. We are recruiting patients meeting all of the following inclusion criteria: (1) histologically confirmed malignant tumor or clinically confirmed neoplastic lesion that is speculated to be appropriately treatable by resection; (2) scheduled to undergo highly invasive surgery, defined as tumor resection estimated to last 6 h or longer [39]; (3) Hospital Anxiety and Depression Scale-Anxiety (HADS-A) score of at least 1 at the time of registration; (4) ability to take study drugs orally; and (5) age of 20 years or older.

Exclusion criteria are as follows: (1) diagnosis of delirium based on the Diagnostic and Statistical Manual of Mental Disorders, Fifth Edition (DSM-5) at the time of recruitment; (2) use of any Japanese herbal medicine within 4 weeks before the day of registration; (3) history of allergy to Japanese herbal medicine; and (4) hypokalemia rated as grade 2 or higher according to the Common Terminology Criteria for Adverse Events, fourth version (CTCAE v 4.0) within 8 weeks before the day of registration.

The subject accrual period is from August 2017 to March 2019, and we plan to complete the analysis by March 31, 2021.

### Registration and randomization

**Fig. 1:**
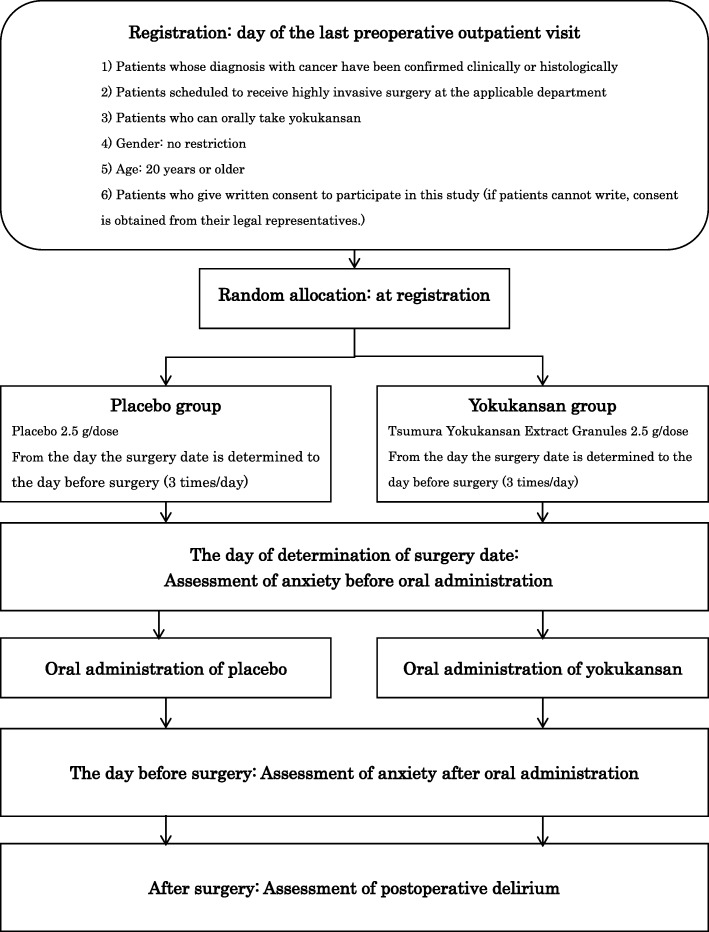
Flowchart of the study procedure

**Fig. 2:**
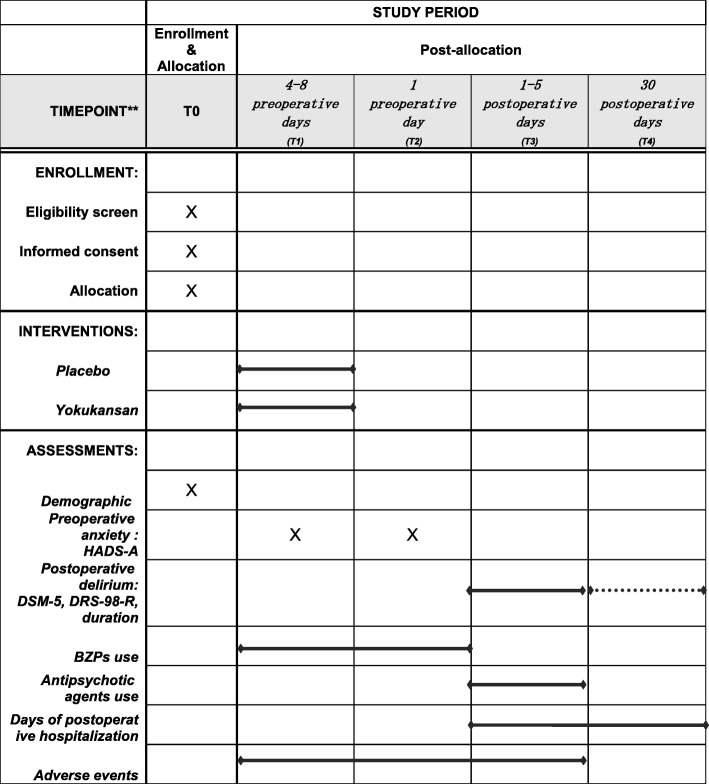
SPIRIT figure: schedule of enrollment, interventions, and assessments. *HADS-A* Hospital Anxiety and Depression Scale-Anxiety, *DSM-5* Diagnostic and Statistical Manual of Mental Disorders, Fifth Edition, *DRS-R-98* Delirium Rating Scale-Revised-98, *BZP* benzodiazepine

The investigators are conducting a screening survey of patients scheduled to undergo highly invasive surgery, as specified in the inclusion criteria. The outline of this study is shown in Figs. 1 and 2. The Standard Protocol Items: Recommendations for Interventional Trials (SPIRIT) checklist is provided as Additional file 1.

Because the drugs are indistinguishable in appearance, the study is blinded to the person responsible for study drug allocation. Eligibility is confirmed at the time of registration, and treatment groups are randomly allocated. No stratification factors are used. The investigators are not informed in detail about the random allocation procedures. The subject registration and random allocation of treatment groups are conducted by a Web-based system. Randomization was balanced with randomly permuted blocks and implemented with an interactive Web-response system which assigned a unique code that dictated the treatment assignment and matching study drug kit for each patient. Thus, treatment assignments were masked from patients and study personnel. Prior to data analysis, the allocation table is retained by the person responsible for studying drug allocation. We have not set criteria for discontinuing or modifying allocated interventions for a given subject.

### Intervention

From the day the surgery date was determined to the day before surgery, one packet of the study drug is administered orally three times a day, before or between meals. The intervention group receives Tsumura Yokukansan Extract Granules (TJ-54, Tsumura & Co., Tokyo, Japan). The daily dose of 7.5 g contains the following herbal medicines: Atractylodes Lancea Rhizome 19.5%, Poria Sclerotium 19.5%, Cnidium Rhizome 14.6%, Uncaria Hook 14.6%, Japanese Angelica Root 14.6%, Bupleurum Root 9.7%, and Glycyrrhiza 7.3%. The placebo group receives granules prepared from lactose and other ingredients not containing Yokukansan extract powder, with an appearance and taste formulated to be as similar as possible to those of Yokukansan.

The treatment period is specified as 4 days or longer prior to the surgery. Compliance is rated on the following 3-point scale: (1) at least two-thirds of the dose was taken, (2) at least one-third but less than two-thirds of the dose was taken, and (3) less than one-third of the dose was taken. We prohibit the use of glycyrrhizin and other herbal medicines for 4 weeks before administration of the study drugs.

The HADS-A is used to assess anxiety before and after administration. Subsequently, delirium is assessed daily using the DSM-5 from 1 to 5 days postoperatively. In subjects with delirium only, the severity of delirium is rated using the Delirium Rating Scale-Revised-98 (DRS-R-98), and the duration of delirium is assessed until it resolves or until 30 days after surgery, whichever is later.

### Primary outcomes

#### Change in preoperative anxiety (change in HADS-A score)

Preoperative anxiety is assessed using the HADS-A [40, 41]. The HADS is a self-reported measure of mental status related to depression and anxiety and consists of 14 questions in total: seven pertain to depression (HADS-D), and the remaining seven are about anxiety (HADS-A). Subjects are asked to respond to HADS-A questions on a 4-point scale from 0 to 3; thus, the maximum score is 21. A higher score indicates worse anxiety. In this study, the efficacy of the study drug is assessed by the difference in the HADS-A score before and after oral administration. Researchers ask for the HADS-A results by phone before Yokukansan or placebo is administered for the first time, and they assess the second HADS-A score in the afternoon the day before surgery.

#### Incidence of postoperative delirium

The proportion of patients diagnosed with postoperative delirium is calculated among those in whom planned surgery is completed. In this study, trained psychiatrists or clinical psychologists assess postoperative delirium on a daily basis using DSM-5 [6] within 5 days after surgery. The DSM-5 offers a standard criterion for the classification of mental disorders. Many mental health professionals use the manual to determine a patient’s diagnosis and to communicate this diagnosis to other practitioners.

We clinically demonstrated and discussed our findings on postoperative delirium before this study, with a final determination made once there was satisfactory agreement between the researchers and the principal investigator (SW) regarding the diagnosis of delirium. The researchers assessed patient samples simultaneously and independently to check the inter-rater reliability of the DSM-5 (20 times for each researcher).

### Secondary outcomes

The secondary outcomes are severity score of postoperative delirium assessed by duration of postoperative delirium, amount of benzodiazepines used before surgery, amount of antipsychotic agents used after surgery, and number of days of postoperative hospitalization. The severity of delirium is assessed using the Japanese version of DRS-R-98 [42, 43]. Although this scale rates delirium using the sum of two subscales—the severity score, based on the evaluation of 13 items, and the scores for three items related to diagnosis—this study used only the severity score. Each item is scored from 0 to 2 or 0 to 3, with a higher score indicating a more severe condition.

### Safety outcomes

We define adverse reactions as any undesirable or unintended signs, symptoms, or diseases that occur from the day a study drug is administered through 5 days after surgery. We do not consider events to be adverse reactions if they are judged by the participating surgeons to be related to the surgery.

We describe the details of serious adverse events. The number of cases and the rate are calculated for each study group based on the CTCAE v 4.0. Regarding clinical laboratory values, we calculate summary statistics of changes from actual values and pre-dose values for each treatment group.

### Sample size calculation

In this study, two primary endpoints are specified. If the superiority of Yokukansan over placebo for either endpoint is confirmed, the drug would be considered to be effective. Therefore, considering the Bonferroni adjustment for multiplicity, a two-sided significance level of 2.5% is used for each endpoint. Regarding the incidence of postoperative delirium—one of the primary endpoints—a previous meta-analysis reported that antipsychotics reduced the incidence of delirium by 50% compared to placebo [44]. The effect of Yokukansan against BPSD, which is thought to be a condition similar to delirium, has repeatedly been demonstrated in randomized controlled trials and is non-inferior to that of antipsychotics [27, 30–34], so we anticipated obtaining the same results against postoperative delirium. Therefore, we estimated an effect size for Yokukansan of 0.5 for postoperative delirium. Yokukansan has few side effects, and if we can show that its effects are comparable to those of antipsychotics, the results may change current clinical practice, in which antipsychotics are generally used as a first line pharmacotherapy to prevent postoperative delirium. Based on an observational study that we performed using exactly the same inclusion and exclusion criteria at the same hospital [5], we estimated the incidence of delirium at 40% in the placebo group and 20% in the Yokukansan group. Given a two-sided significance level of 2.5% and a statistical power of 80%, this indicates that 99 subjects per group are necessary. As for the change in preoperative anxiety, the minimum clinically meaningful difference in anxiety score was shown in a previous study to be 1.32 points, although in that case the subjects were patients with chronic obstructive lung disease [45]. Therefore, in this study we assume a between-group difference in the mean change in preoperative anxiety of 1.5 points. Assuming a 3-point standard deviation in the preoperative anxiety score, a two-sided significance level of 2.5%, and a statistical power of 80% based on the results of an observational study conducted at the National Cancer Center Hospital [13], 78 subjects per group are necessary. Considering the above and speculating that some subjects may later be found to be ineligible or may withdraw from the study, the planned number of registered subjects was determined to be 110 per group, or 220 in total.

### Statistical analysis

The analysis sets for efficacy and safety include all registered subjects. The mean change in the HADS-A score before and after oral administration in each group, the point estimate of the between-group difference, and the 97.5% confidence interval are calculated. Group means are compared using the *t* test. The incidence of postoperative delirium in each group, the point estimate of the between-group difference, and the 97.5% confidence interval are calculated, and the proportions are compared by the chi-square test. Because there are two primary endpoints, Bonferroni adjustment for multiplicity is performed, and a two-sided significance level of 2.5% is used for the two comparisons. If superiority of the Yokukansan group is observed in either of the endpoints, treatment with Yokukansan will be considered to be useful. The interim analysis examined only the Yokukansan group but not both groups, and we do not regard this as a multiple analysis.

Interim analysis will be performed when the number of subjects who have completed the protocol treatment and are evaluable for both endpoints reaches one-third (*n* = 74) of 220, which is the target number of registered patients in this study. At that time, we will determine whether or not continuation of the study is appropriate. If it is determined that the main purpose of the study has not been fulfilled, the study will be discontinued, and the study results will be promptly disclosed. In principle, registration will not be suspended during the interim analysis. When the point estimate of the mean preoperative anxiety score in the Yokukansan group exceeds 5 points or the incidence of postoperative delirium exceeds 40% in the interim analysis, the Independent Data Monitoring Committee, which consists of three external members, will judge whether or not to discontinue the study.

Serious adverse events are described in detail. For other adverse events, the number of subjects with the highest observed grade and the incidence of the highest grade in each treatment group are calculated, and the groups are compared by the Mantel test.

We define the analysis sets as follows. The *full analysis set* consists of all randomized subjects who satisfy eligibility criteria, receive any study drug, participate in at least one postbaseline assessment, and have no Good Clinical Practice (GCP) violations. The *per-protocol set* excludes subjects in which the study is not carried out as per protocol because consent is withdrawn, whose surgery is canceled, who do not take the study drug according to the protocol, etc. The *safety analysis set* includes all subjects who receive any study treatment (including control) but excludes subjects who withdraw prior to receiving any treatment. Data that have been rejected due to missing information, lack of clarity, or faulty handling of data are not included in the analysis. We do not impute the missing data.

### Quality control and assurance

The study-related division of the sponsor, Tsumura & Co., performs quality control. Clinical research associates decide on issues related to data entered into the electronic data capture system and report their conclusions to the investigator. If any problems are anticipated, the investigator takes the appropriate measures.

To determine whether the study is being conducted in accordance with the protocol and the GCP, the GCP audit division of the sponsor performs a GCP audit and formulates an appropriate plan following the Tsumura-GCP audit procedure.

The GCP audit division of the sponsor performs GCP audits of all facilities involved in this study, including the study site and facilities at external organizations and contract research organizations, at appropriate times during or after the study.

### Research ethics approval

This study was designed and is undertaken in compliance with the Declaration of Helsinki and the “Ethical Guidelines for Medical and Health Research Involving Human Subjects” (Public Notice of the Ministry of Education, Culture, Sports, Science and Technology and the Ministry of Health, Labor and Welfare). All study protocols, informed consent forms, and other requested documents were approved by the Institutional Review Board and the Ethics Committee of the National Cancer Center in Japan.

The investigators sufficiently explain the details of the study to the subjects in accordance with the informed consent documents and obtain subjects’ voluntary written consent to participate in the study. After provision of consent, subjects are withdrawn from the study according to the following criteria: (1) if the subject withdraws consent; (2) if the primary disease is exacerbated or an adverse event occurs, and continuation of the study is judged to be difficult; (3) if a subject is found to be ineligible for the study after it begins; and (4) if a subject discontinues the study treatment at his or her own discretion.

The documents or records that should be stored at the study site, including those related to subject consent, those on which case report forms are based, records of deliberation by the review board, documents related to requests and contracts for the study, and the management table for study drugs, will be stored for 3 years after the discontinuation or completion of the study. After the storage period, the documents and records will be anonymized and discarded by an appropriate method. We do not plan to grant public access to the full protocol, participant-level dataset, or statistical code.

### Funding source and conflicts of interest in this study

Funding and other resources necessary for the conduct of this study are provided by Tsumura & Co., the sponsor. In conducting and reporting this study, the investigator and coinvestigator must not change their professional judgment for the purpose of obtaining monetary profit or other personal benefits. The investigator and coinvestigators are not related to Tsumura & Co. by employment or personal connections. If patent or related rights arise in association with this study, the study site and the sponsor determine the appropriate right-holders.

The sponsor also provides study drugs and prepares study-related materials, including the protocol and statistical analysis plan, and supports the preparation of the informed consent documents and the form for withdrawal of consent.

The principal investigator is responsible for writing the report and deciding whether to publish it.

## Discussion

Preoperative anxiety and postoperative delirium affect both short- and long-term prognoses in patients with cancer and therefore need to be appropriately assessed, treated, and prevented. However, neither preventive nor therapeutic methods have been established for these conditions.

The present study is the first randomized, double-blind, placebo-controlled study conducted to clarify the effects of a Japanese herbal medicine, Yokukansan, for the prevention and treatment of perioperative psychiatric symptoms in patients with cancer. Although Yokukansan has already been approved for anxiety, existing findings are insufficient to allow a clear conclusion regarding its efficacy in preoperative conditions in particular. There are no standard therapeutic regimens for preoperative anxiety or postoperative delirium in cancer patients. For these reasons, if the present study can clarify the therapeutic effect of Yokukansan on preoperative anxiety, its preventive effect on postoperative delirium, and its safety in cancer patients, it will greatly contribute to the establishment of standard therapies for perioperative psychiatric symptoms in this population, leading to the provision of safer medical care with higher compliance. However, if the effect of Yokukansan is not verified in this study, the lack of clear evidence will suggest that continued use of this agent in daily practice can be stopped.

## Trial status

The protocol version number is Ver 3.2, and the date is September 6, 2017. The trial was initiated on August 14, 2017, with 195 subjects randomized by October 5, 2018. We plan to complete recruitment on March 31, 2019.

## Additional file

Additional file 1: SPIRIT 2013 checklist: recommended items to address in a clinical trial protocol and related documents. (DOC 124 kb)

## Abbreviations

ᅟPSD: Behavioral and psychological symptoms of dementia
CTCAE v 4.0: Common Terminology Criteria for Adverse Events, fourth version
DRS-R-98: Delirium Rating Scale-Revised-98
DSM-5: Diagnostic and Statistical Manual of Mental Disorders, Fifth Edition
GCP: Good Clinical Practice
HADS-A: Hospital Anxiety and Depression Scale-Anxiety
HADS-D: Hospital Anxiety and Depression Scale-Depression
J-SUPPORT: Japan Supportive, Palliative and Psychosocial Oncology Group
